# Implementing ethics reflection groups in hospitals: an action research study evaluating barriers and promotors

**DOI:** 10.1186/s12910-019-0387-5

**Published:** 2019-07-16

**Authors:** Henriette Bruun, Reidar Pedersen, Elsebeth Stenager, Christian Backer Mogensen, Lotte Huniche

**Affiliations:** 1 Focused Research Unit in Psychiatry, Institute of Regional Health Research, University of Southern Denmark, J.B. Winsløws Vej 19,3, 5000 Odense C, Denmark; 2 Focused Research Unit in Emergency Medicine Institute for Regional Health Research, University of Southern Denmark, J.B. Winsløws Vej 19,3, 5000 Odense C, Denmark; 3 Department of Psychology, Faculty of Health Sciences, University of Southern Denmark, Campusvej 55, 5230 Odense M, Denmark; 4 Center for medical Ethics, Institute of Health and Society, University of Oslo, Kirkevejen 166, 0450 Oslo, Norway

**Keywords:** ERG, Implementation, Action research, Evaluation, Psychiatric hospital, Emergency hospital

## Abstract

**Background:**

An ethics reflection group (ERG) is one of a range of ethics support services developed to better handle ethical challenges in healthcare. The aim of this article is to evaluate the implementation process of interdisciplinary ERGs in psychiatric and general hospital departments in Denmark. To our knowledge, this is the first study of ERG implementation to include both psychiatric and general hospital departments.

**Methods:**

The implementation and evaluation strategies are inspired by action research, using a qualitative approach and systematic text condensation of 28 individual interviews and 4 focus groups with clinicians, ethics facilitators and ward managers.

**Results:**

The implementation process was influenced by both structural factors and factors related to clinicians having different values, interests and experiences. Structural barriers and promotors in the process to implement ERG included the following sub-categories: Organizational factors, recruitment and training of ethics facilitators, the deliberation model, planning and recruitment of participants to the ERGs, the support of the ward managers and the project group. Barriers and promotors found among clinicians included the following sub-categories: Expectations and pre-understandings of ERGs, understandings of a physician’s job, challenges experienced by ethics facilitators. At the end of the study, when it was decided that the ERGs should be continued, the implementation strategies were remodeled by the participants to meet new challenges.

**Conclusion:**

The study of ERG implementation identified important structural and professional barriers and promotors that are likely to be relevant to anyone wanting to implement ethics support services across various types of healthcare services.

## Background

Ethical dilemmas are an inseparable part of daily clinical practice in healthcare [1–4]. A moral dilemma is defined by Beauchamp and Childress as *circumstances in which moral obligations demand or appear to demand that a person adopt each of two (or more) alternative but incompatible actions, such that the person cannot perform all the required actions* [1]. In clinical practice, situations involving ethical elements or questions are often not perceived by clinicians in that way; therefore the more pragmatic term “ethical challenge” has been used in this study. An ethical challenge may involve doubt or conflicting interests among clinicians, patients and/or their relatives [3]. Handling ethical challenges in daily clinical practice has been found to cause distress among clinicians [5, 6].

Various types of clinical ethics support services (CESS) have been established in order to assist clinicians dealing with ethical issues. Important examples are clinical ethics committees (CEC) [7], clinical ethics consultation [8], moral case deliberation (MCD) [9] and ethics reflection groups (ERG) [10].

CECs are described by Rosoal et al. [11] as top-down approaches in which an ethicist, a philosopher *or a group of “experts” has an influential advisory role or acts as the primary ethical decision maker, providing advice or recommendations.* In the same paper, bottom-up approaches, such as ERGs and MCD, are described as taking their starting point in *healthcare personnel’s everyday experiences of ethical issues in clinical practice.* Their goal is to stimulate ethics reflection and promote mutual understanding between professional groups, but also between professional perspectives and the perspectives of patients and relatives. Many CECs work along the same lines, i.e. facilitating ethics reflection and raising moral awareness, rather than acting as decision-makers or experts. Clinical ethics consultation, sometimes anchored in CECs, also strives to bridge the strict division between top-down and bottom-up approaches. Clinical ethics consultation is provided by an individual or a small team in the ward, at the request of clinicians.

Although literature has pointed out the significance of top-down approaches like CEC [7], a number of barriers to referring a case have also been registered. Slowther et al. [12] emphasize that low organizational awareness of ethics difficulties and a low perceived need for ethics support may lead to low referral rates. Quoting a CEC member, Pedersen et al. [13] offer another explanation why healthcare professionals refrain from reporting ethics cases to a committee; that submitting might be seen as disloyalty towards colleagues. The paper concludes that within the medical culture there is no tradition for sharing difficult cases with anyone outside the professional community. Discussions related to clinical ethical issues are seen as part of the ongoing practice in hospitals, whereas submitting a case to a clinical ethics committee may be seen as a formalization of the involvement of “outsiders”.

Among others, Lillemoen et al. [10, 14–18] describe the significance of bottom-up approaches like ERGs and MCD. Although scarce, challenges in implementing this kind of CESS have been described [19–23]. Reiter-Theil describes 10 tasks and challenges when initiating and maintaining clinical ethics support in psychiatry: *Suggestions I–III are of organisational nature, IV–X have explicit ethical content concerning the ethos or professionalism of the ethics consultant and CES practice* [20]. In a recent literature review, Haan et al. [24] focus on the research question: What is the impact of moral case deliberation on groups of healthcare professionals in clinical settings? They describe some facilitators and barriers to consider when preparing MCD: A facilitator capable of creating a safe and open atmosphere, a concrete case, the commitment of participants, a focus on the moral dimension, and a supportive organization.

The field of CESS is relatively young in Denmark. As one of the first in Denmark, the CEC for Psychiatry in the Region of Southern Denmark was established in 2010. To expand ethical reflection from the CEC to include everyday clinical practice, it was decided to implement ERG. In this research project, an ERG is an ethics facilitator-led deliberation process organized in an interdisciplinary group of clinicians. The participants are invited to reflect on a specific ethical challenge from everyday clinical life. As in the CECs in the Region of Southern Denmark [25], a modification of the SME model (a deliberation model developed at the Center for Medical Ethics (“SME” in Norwegian) at the University of Oslo) [7, 10] was used as reflection model in the ERGs. The SME model centers on the following questions: What is the ethical problem? What are the facts of the case? Who are involved, and what are their views? What values, laws and guidelines are relevant? What are the alternative courses of action? To secure continuity and facilitate planning, each ERG met twice a month and each meeting was scheduled to last 45–60 min. The participation of ward managers was optional.

### The aim of the project

This paper is the result of a research project inspired by the work of a CEC in psychiatry in the Region of Southern Denmark and an analysis of which ethical challenges clinicians in mental healthcare experience and discuss with a CEC [25]. By exploring barriers and promotors in the implementation process, along with the overall perceived significance of ERG from the perspectives of clinicians, ethics facilitators and ward managers, new insights are sought in order to further qualify the development of ERG implementation strategies.

The aim of this paper is to evaluate the implementation process of interdisciplinary ERGs in psychiatric and general hospital departments in Denmark. The research process has been guided by the following research question: What constitutes barriers to and promotors of successful implementation of ethical reflection groups in an emergency department and two psychiatric wards in a Danish hospital?

## Methods

Implementation of ERGs in a hospital setting is a complex intervention. The research approach chosen for this project was derived from action research [26]. This research strategy is found to be well suited for implementation, investigation and evaluation of complex healthcare interventions aiming to involve researchers and clinicians in the research process, and when existing research is limited. The study was designed and carried out in accordance with the action research cycle described by Malterud [27] for practical application in medical research. The action research cycle consists of the following phases: 1. Identify the problem; 2. Summarize previous experiences; 3. Determine the aim of the intervention; 4. Plan and develop the intervention method; 5. Design and articulate the intervention strategy; 6. Implement the action and evaluate experiences and results, and 7. Redefine the problem.

### Implementation strategies

The implementation strategy was based on previous experiences and studies implementing clinical ethics support [20, 22], focusing on bottom-up approaches such as moral case deliberation [19] and ERG [21]. Central implementation strategies (see Table 1) were: Formation of a cross-site project group holding one-day and mini-courses for ethics facilitators, and later on training and peer supervision of ethics facilitators. Table 1 gives a description of the implementation strategies.

**Table 1:** Implementation strategies

The project group	- Formed after selection of project sites and ethics facilitators - Members: The first author, managers at all project sites, all ethics facilitators - Three meetings and an “end-of-study workshop” were conducted
One-day course for ethics facilitators	- Planned as a standard course once a year by the Clinical Ethics Committee of Psychiatry - The program topics: • What is structured ethics reflection? • What is an ethics dilemma or challenge? • Introduction to ethics principles and positions • Introduction to the SME model (see below) • How to facilitate an ethics reflection group • Workshop; in smaller groups the participants practiced and used the SME model on an ethics dilemma experienced by one of the course participants
Mini-course for ethics facilitators	- A tailored short-version of the one-day course for one or two participants - The workshop element was omitted
Training for ethics facilitators	- Training of ethics facilitators in using the SME model in their ethical reflection groups - 6–8 meetings in each ethics reflection group
Supervision	- Supporting and supervising the ethics facilitators in using the SME model - 3 meetings in each ethics reflection group

Obviously, an important cornerstone in the implementation process was education and training of the ethics facilitators. The mini-course was developed because it turned out to be impossible for all ethics facilitators to participate in the-1 day course for ethics facilitators held once a year. Also, the training needed to be further developed during the study as ethics facilitators pointed out that at the time of their first meeting in their ERG, they had at most experience from the workshop conducted at the one-day course. Thus, previous experience [28, 29] inspired the education and training procedure.

### Selection of participating departments

At the beginning of the project, the implementation strategies were discussed with the heads of the psychiatric and the emergency departments. The head of the emergency department decided that the whole department should participate. As the psychiatric department was bigger, consisting of six decentralized inpatient wards and six outpatient clinics, it was decided for one inpatient ward and one outpatient clinic to participate. In order to give the ward managers a basis for deciding whether to participate or not, the project was introduced to all of them. Almost immediately an inpatient ward announced that they wished to participate. Referring to time constraints, two outpatient clinics declined participation, but one consented to participate. Table 2 gives a description of participating departments.

**Table 2:** Description of participating departments

	Site I	Site IIA	Site IIB
Name	Emergency department	Psychiatric inpatient ward	Psychiatric outpatient clinic
Characterisation of patients	Patients in need of urgent general or psychiatric medical attention	Patients experiencing deterioration in psychiatric disorder	Patients living at home, receiving specialized psychiatric assistance
Subdivision of sites	Two wards: 1. Quick assessment, patients stayed for hours 2. Patients stayed for few days	Patients were admitted for days or weeks	Three teams divided according to diagnosis, patients were admitted for several months
Number of beds	38	32	none
Educational background of staff	Senior physicians, junior physicians, nurses, auxiliary nurses	Psychiatrists, junior physicians, psychologists, social workers, physiotherapists, nurses, auxiliary nurses	Psychiatrists, psychologists, occupational therapists, social workers, nurses, auxiliary nurses
Experience with ethic reflection	–	–	+
Involved in other quality of care projects	–	+	–

The ward managers decided to organize the ERG differently at the three sites: At Sites I and IIA, open interdisciplinary groups were organized, and all clinicians working on the day of the meeting were invited. At Site IIB, the ward manager decided that the group should be made up of two standing members from each team, while all other clinicians were invited to participate on an ad hoc basis.

### Data collection

Data was collected during the entire research period. Different research methods were used:

#### Participant observation

During completion of the implementation strategies, participant observation was carried out. Participant observation can be conducted with varying degree of researcher participation [30]. In this project, the researcher was involved in all implementation strategies; therefore a position as passive observer was ruled out. Instead written summaries of meetings in the project groups were made. After the training of ethics facilitators, descriptions, reflections and considerations were made on audio file. Except for one (a technical error), all supervisions of ERGs were tape-recorded, as was the end-of-study workshop.

#### Individual interviews

Participants were chosen strategically and with a focus on variety [31]. Participants expected to be able to give “thick descriptions” were chosen. [32] The purpose was to gather knowledge about the implementation of ERG seen from the perspective of different clinicians, differently engaged in the ERGs. Table 3 shows the educational background and the type of engagement in the project of each participant individually interviewed.

**Table 3:** Participants in individual interviews

Role in ERG	Educational background						
	Physician	Nurse	Psychologist	Occupational therapist	Radiographer	Auxiliary nurse	In total
Ward manager	1	4					5
Ethics facilitator		5	1				6
Participants	3 + 1 student	5 + 2 students	1	3	1	1	17
In total	5	16	2	3	1	1	28

Due to the action research methodology, the researcher was involved in both implementation and evaluation of the ERGs. As a consequence, participants might be reluctant to express criticism. To overcome this challenge, critical participants and perspectives were actively requested, and during the individual interviews criticism specifically asked for and examined.

Interviews were carried out based on a semi-structured interview guide focusing on the three overriding issues: 1) What is daily practice in the department? What kind of ethics challenges are there? 2) How was the ERG implemented in your department and what is your experience of facilitating/participating in the ERG? 3) What is the significance of the ERG for daily practice? The individual interviews lasted an average of 49 min, ranging from 31 min to 65 min.

#### Focus groups

In focus groups, the interaction between participants triggers more expressive and responsive viewpoints, imparting new knowledge and a different angle on the ERG and the implementation of it. Due to time constraints, the ward managers were involved in the organization of the focus groups. At some sites, already planned staff meetings were used. Table 4 shows the organization and the educational background of the participants in the focus groups.

**Table 4:** Participants in focus groups

Focus group	Number of participants	Staff meetings	Educational background
Site I	5		Nurses (5)
Site IIA	10	X	Auxiliary nurses (3), nurses (6), physiotherapist (1)
Site IIB	17	X	Auxiliary nurses (3), Nurses (8), psychologists (2), occupational therapists (3) social workers (1)
Ethics facilitators	4		Nurses (3), psychologists (1)

The first author was moderator in all focus groups, and the meetings were structured according to the above three overriding issues framing the individual interviews. The focus groups lasted an average of 43 min, ranging from 36 min to 52 min.

All interviews, including the individual interviews, were tape-recorded and transcribed verbatim. During the transcription and additional processing of data, great care was taken to anonymize persons and places. In the results section, the pronouns *he* or *she* are used at random.

### Analysis

The analysis was mainly based on the individual interviews and the focus groups. The participant observations were used as background data. As an analytic strategy, a systematic text condensation has been used [33]. NVivo 11 was used to systematize the data during the examination. All individual interviews and focus groups were read by the first author. The analytic approach was abductive, involving moving back and forth between the transcripts and theoretical concepts. First, an inductive focus was applied: Getting an overall impression, starting to make categories based on the overall themes in the transcripts [32]. In that process, no significant differences between general and psychiatric departments were found. During dialog with the other authors, the categories were processed and rearranged. Later, as the analysis was informed by theoretical concepts and literature, a more deductive approach was applied. Action research theory, identifying the behavior of persons as inevitably related to both the environment and the way in which the person perceives the situation, informed the analyses, as did relevant literature [10, 13, 19].

The analytic focus in this article is on barriers and promotors in the implementation process. They are related to structural factors and to the clinicians involved. However, some elements are associated with both barriers and promotors. A good example of this is the recruitment and training of ethics facilitators: Ethics facilitators promote the implementation of ERG, but there are several barriers to both recruitment and training. Therefore it can be difficult to separate barriers from promotors [34]. As a consequence, the overall analytic categories are: 1) Structural barriers and promotors in the implementation process, and 2) barriers and promotors found among clinicians. Table 5 shows the analytic process.

**Table 5:** The analytical process

Categories	Sub-Categories
Structural barriers and promotors of the implementation of ERG	• Organizational factors • Recruitment and training ethics facilitators • The deliberation model • Planning and recruiting participants to the ERG • The support of head of the ward • The project group
Barriers and promotors found among healthcare professionals	• Expectations and pre-understandings of ERG’s • Understanding of physicians´ job • Challenges experienced by ethics facilitators

## Results

The results of the analysis will be presented in three sections. Firstly, structural barriers and promotors in the implementation of ERG will be presented. The structural elements are both preexisting administrative and financial circumstances, but also the implementation strategies themselves are seen as new structural elements in the wards. Secondly, the barriers and promotors found among clinicians will be presented. This section describes how pre-understandings, attitudes and interests among various participants influence the implementation process. Thirdly, the new initiatives and changes based on the results and experiences of this study will be presented.

### Structural barriers and promotors in the implementation process

#### Organizational factors

Lack of time was presented as a general barrier. A facilitator even said that when recruiting participants, time constraints posed a dilemma:

*[ ] but there is also a dilemma here, because it is at the expense of not seeing their patients. So honestly, if it’s a really busy day, it’s a dilemma for me as the facilitator, because if I ask the physicians to come to our meeting, then there is nobody left to do the rounds and there’s no flow in the unit. There have been a few times when I haven’t asked the physicians to come because I thought it would be better if they saw patients, especially if patients were doing badly (Ethics facilitator focus group).*

Administrative factors and the financial management of the work performed by clinicians constituted different kinds of barriers. At Site IIB, the activities of individual clinicians were evaluated through registration of the number of patient consultations accomplished during a certain amount of time. Also, some of the funding of the department was allocated on the basis of the overall number of consultations with patients. Clinicians were well aware that this registration system was a part of their working conditions. When ERGs were introduced, there was no reduction in the number of consultations expected to be delivered by each clinician. Thus, the ERG was added to their existing workload. The ward managers experienced some opposition to the ERG among clinicians, who, referring to the patient consultation requirements stated that they could not find the time for yet another staff forum.

When organizing an interdisciplinary initiative like the ERGs, the ethics facilitators experienced the administration of the working schedules of different groups of clinicians as a barrier. One facilitator pointed out difficulties in getting sufficient insight into the working schedules and daily routines of physicians. As a consequence, it was difficult to find a time convenient for all clinicians.

At Site IIA, the abovementioned engagement in another projects caused difficulty in finding a suitable time for the meetings in the ERG. As a result, they only managed to have 9 meetings after the completion of the training period, which was quite few compared to Site I and Site IIB where they managed 19 and 21 meetings respectively in the same period.

#### Recruitment and training of ethics facilitators

Ethics facilitators played a key role in the ERG, both in organizing it and in facilitating the ethics deliberation process. Recruitment of clinicians to act as ethics facilitators depended on prior knowledge and experience with ethics reflection. At Site IIB, some clinicians already had experience and knowledge about structured ethics reflection. Moreover, some had been involved in the work of the CEC of Psychiatry. Here, the recruitment of ethics facilitators was easy and straightforward. At Site I, there was little or no knowledge, and here it was difficult to engage any clinicians. In the end, an experienced clinician agreed to take on the role at the beginning of the project. As the ERG became well-known at Site I, other clinicians signed up to be facilitators.

There was an overall positive response to the one-day course for ethics facilitators. Still, ethics facilitators participating in either of the two courses held requested further introduction to ethics theories and practical experience in a protected setting, before having to facilitate an ERG in their own ward. As for the training subsequently introduced, they reported afterwards that it was mainly through this implementation strategy that they learned how to act as ethics facilitators. Another positive consequence of this training was that the participants were introduced to the ERGs in a more qualified way.

When the courses and training were finished, there was still some insecurity among the ethics facilitators. Some reflected on their role as ethics facilitator, comparing it to well-known roles as for example teaching or peer supervision. However, to a certain extent, there was a disproportion between the insecurity described by the ethics facilitators and the insecurity perceived by the participants. Apart from one participant, who said she experienced a drop in quality towards the end of the training, there was widespread support and appreciation of the job performed by the ethics facilitators. Over time, the ethics facilitators became more experienced and accustomed to their role. One said that she slowly realized that her role was mostly that of a process ward manager. Another said that her performance anxiety decreased once she realized that it was in fact the participants of the group who were to do the reflection, not her as the ethics facilitator.

#### The deliberation method

The ethics facilitators described the SME model, as well as the fact of being two facilitators in each group, as supportive. In order for the participants to stick to the SME model during the deliberation, a whiteboard for cues and summations were used. When the dialog sometimes got off track, the facilitators used the SME model to adjust the reflection process.

During the project, the ethics facilitators increased their understanding of the SME model, and they experienced that it was useful for handling some of the challenges described below. They reported more self-confidence, for example selecting a specific ethics challenge experienced by one of the participants instead of the more general cases, and even more ambition in their job as ethics facilitators. One ethics facilitator said that she would have liked to go through all the bioethics principles [1] instead of just the principle of autonomy, the only principle they managed to reflect on at the beginning. Moreover, the structure of the SME model was used to communicate the deliberation process; either as a photo of the whiteboard for cues and summations or as a written summary.

#### Planning the ERG and recruiting participants

In cooperation with the ward manager, the ethics facilitators organized the ERGs. The structure of the groups influenced the number of initiatives necessary to recruit participants among other clinicians. At site IIB, the decision to appoint standing members reduced the need for initiatives to recruit participants. The ethics facilitators at Sites I and IIB took a number of organizational initiatives. After consulting the staff work schedule, they wrote a personal e-mail inviting staff at work to participate. On the day of the meeting, they reminded clinicians about the meeting. Taking into account the current workload, it was specified who should participate in the meeting. During the project period, it became evident to the ethics facilitators that information about the ERG failed to reach clinicians working according to different work schedules than the ward staff schedule. This issue was solved by emailing psychiatrists, physicians, social workers and psychologists directly.

#### The support of the ward managers

Crucial to the success of the implementation was the support of the heads of departments and the ward managers at each project site. The encouragement of the ward managers was important in two regards: 1) To support the ethics facilitators in their own training and organizational tasks, and 2) to support and encourage other clinicians to participate in the ERG.

At all three project sites, the ward manager entrusted the organizational work to the ethics facilitators. The ethics facilitators took on the task, but at the same time they said that they were dependent on support from the ward manager. The ward managers were all well aware of their importance for the success of the implementation process. All the same, there was variation across the sites as to how often the ward manager participated in ERG. One said that the project had his attention and that he, as a representative of the management team, should lead the way and reserve time to participate in the ERG. Another ward manager underlined the importance of not participating, as it left more room for the clinicians to deliberate. Yet another supported the ERG by undertaking the work duties of the participants while they attended the meeting. One ethics facilitator described how a very engaged ward manager would ask her about the progress of the group. To her, this was proof of the involvement of the ward manager.

At Site IIB, the management of healthcare activities by registration of patient consultations created some resistance among clinicians. They asked the ward manager if participation in the ERG was voluntary, and he answered:

*Oh no, this is not voluntary, it’s something we have to do. We have chosen to sign up and participate, so we are going to have an ERG in our unit [ ] And it is one of my focal points as head of the unit, and I want those who haven’t participated yet to take part as well (Nurse in individual interview).*

By this quote, the ward manager showed his support and stressed the importance of the ERG by stating that he expected all clinicians in his team to participate.

#### The project group

Both the ward managers and ethics facilitators mentioned the cross-site project group as an important forum during the implementation process. It was used for mutual inspiration, both concerning ethics facilitator insecurity and how to manage it, but also for sharing ideas on how to manage the organizational tasks and recruitment of participants. Also the ward managers were able to share experiences and inspiration.

### Barriers and promotors found among clinicians

#### Expectations and pre-understanding of ethical reflection groups

Most clinicians welcomed the implementation of ERGs. Interest in, knowledge about and experience among clinicians acted as promotors in relation to the implementation process. Several years before the implementation of ERGs, a clinician at Site IIB involved in the CEC of Psychiatry had arranged and facilitated meetings in the ward, using the SME model on ethics challenges experienced by clinicians in the ward. Some clinicians still remembered these meetings positively and said that the analysis had been useful to them.

*Some of the older staff members remembered that they had been involved in such a process before and that it had been fantastic, so in that way I think the idea had already been sold even before I started selling it. [ ] Yes, there were some who supported the idea immediately. Some of the older nurses. [ ] So I’m thinking they had a shared history here, something that had already prepared the ground for the idea beforehand* (***Psychologist*** *in individual interview).*

Among clinicians without experience of ethics reflection, several participants mentioned that there might be some reticence or reluctance to participate in the ERG. One said that the name “ERG” had a diffuse ring to it. Another said that before participating, she had the pre-understanding that:

*I thought, this is going to be about really big really vague problems that are neither here nor there [ ] that’s what you think ethical and philosophical deliberation means. Going round and round in circles for what feels like for ever [ ] so what’s been a huge surprise is that it has actually helped me, at a very specific everyday level (Occupational therapist in individual interview).*

For some, the term Ethics Reflection Group had an air of an exclusive club. Some even worried that the ERG might divide clinicians into those who were reflective and competent and those who were not, creating a trendsetting elite regarding ethics issues in the ward. It was also mentioned that there could be a risk that some clinicians might use the ERG as a forum for airing complaints indirectly, or that the group might be dominated by clinicians wishing only to address their own favorite topics. One was afraid that undesirable divisions could emerge, e.g. a group of nursing staff agreeing in a one-sided way that a physician was acting totally unethically. Another feared that some clinicians might avoid participation because they worried about criticism and increased external scrutiny of their actions.

However, more participants described how such pre-understandings had been revised once they had participated in the ERG. Pre-understandings thus represent a barrier to the implementation of ERGs that calls for attention and active handling. In the words of one participant:

*I think it would be great if everybody tried it. That would, you know, help de-mystify it. Because once people have tried it, they’ll know it’s really very down to earth (Psychologist in individual interview).*

Hospitals are hierarchic organizations; therefore support of high-ranking persons like physicians is considered important for successful implementation of interdisciplinary ERGs. Although physician participation was favored by other clinicians, it generally proved difficult to recruit physicians to participate in the ERGs. There were diverse pre-understandings of ethics reflection among physicians, and therefore also varying degrees of motivation and engagement.

One physician said that it would be a good idea to convince as many physicians as possible to participate in the ERG. He elaborated on his statement by saying that physicians are not always aware of the impact of their decisions on people’s lives. And physicians may not always realize the full range of considerations at stake.

Another physician said that in her opinion the ERG provided an opportunity to introduce young physicians to an important part of the daily clinical considerations.

*Because they [the young physicians] arrive straight out of university, armed with their emergency room manual and their heads full of professional knowledge, and now they have to learn to see the human side of things and use that in different situations. And I think it’s important to help them, to step into that new role with them and guide them (Physician in individual interview).*

One senior physician recalled an incident she experienced as a junior physician which had challenged her personal ethics. She was stunned by her own minimal effort in trying to influence the situation, and that as a young physician she *“had become so hardened already that I thought, well, that’s just part of being a physician”.* It was her reflections on this incident years ago and her fear of becoming a cynical physician that motivated her to support the ERG.

Yet another physician said that there was no need for ERGs because in his opinion there were no ethics challenges in daily clinical life. The argument supporting this point of view was that applying informed consent constituted his overall point of orientation.

Moreover, a distinct separation of the roles of physicians and nurses served as an argument to explain why ERGs were a good idea and suitable for nurses – but not for physicians. Although with varying intensity, a distinction between the two professions was repeated several times. It was said that nurses interacted with patients and relatives, while physicians addressed more factual issues, such as the planning and evaluation of treatment. Thus for physicians, ethics was not really relevant.

Conversely, one physician explained how he happened to learn about the existence of the ERG only by coincidence. Noticing that a group of nurses were leaving the ward, he asked where they were going. A nurse told him about the ERG and when he expressed his wish to participate, the nurse was clearly surprised. She had not expected it would be possible to persuade a physician to participate in a group organized and facilitated by a nurse. This conversation made the physician conclude that nurses refrain from inviting physicians to this kind of interdisciplinary teamwork because they are prejudiced against physicians and their wish to participate.

#### Understandings of a physician’s job

Although more evident at Site I, the abovementioned distinction between the approaches of physicians and nurses was noted by nurses from all three project sites. A psychiatric nurse said that some physicians had great difficulties in involving elements that were not based on evidence.

*Well, I think that all this about looking at a patient as a human being, with all his or her problems in life, instead of focusing on fault-finding and trouble-shooting, you know, drawing on softer values as well. And I think our head physician is really good at doing exactly that, but many of our other physicians don’t do that (Nurse in individual interview).*

A nurse said that an overall one-sided focus on evidence made it difficult for physicians to participate in ERGs.

*Yes, physicians are really obsessed with everything being evidence-based, they want valid and reliable data for everything. But that’s not available at the individual level, faced with a specific person. Then it’s a matter of personal priorities if that person chooses to do one thing or another. [ ] It’s a different set of values. And this is where I’ve often experienced that many physicians feel completely out of their depths and then the dialog stops*. *(Nurse in individual interview).*

She also addressed the power relationship between the two professions, especially the expectation that the final decision was made single-handedly by the physician.

*After all, they are at the top of the hierarchy, so no thank you, they don’t want to lose face if they don’t have a definite answer to offer. [ ] Traditionally, the physician has the final word when it comes to treatment. (Nurse in individual interview).*

She said that many physicians are trained to act, and even when they listen *you can’t help sensing their insecurity when they don’t get the final say.*

A physician underpinned this description by saying that the role as a physician carries entirely different obligations and responsibilities, and therefore physicians in general are more closed off to open deliberation than nurses. Some are almost made out to be immune to doubt at all:

*They [the physicians] are the ones who make the decisions, historically it’s always been like that, it’s part and parcel of the job description when you’re a physician. And I’m expected to live up to that (Physician in individual interview).*

This authority to make decisions, the physician went on, makes it difficult for physicians to acknowledge that they themselves are vulnerable, and that they are sometimes in doubt about what to do. It is difficult for physicians to imagine themselves in a situation where they would feel the need to reflect on difficult ethical decision situations together with other clinicians.

Also, it was mentioned that other clinicians, patients and people in general expect physicians to have an answer to every question. Likewise, a physician said that when she asked her senior psychiatrist about his absence from the ERG, he answered that his participation would create an expectation among other clinicians that he had the ultimate answer.

When physicians predominantly concern themselves with specific elements of treatment, a physician said, “*they remain on top of things”*. Participating in the ERG implies that feelings and personal ethics would come into play. He concluded that by staying in their familiar role, physicians managed to keep a distance to that part of their job. A nurse pointed out that physicians are unaccustomed to the working methods of ERGs.

*It’s an unusual situation to be in. It’s takes some time to get used to, you have to drum up the courage to show that you’re uncertain, insecure, in such a dialog. I don’t think that’s something physicians are used to. (Nurse in individual interview).*

A physician agreed by saying that interdisciplinary ERGs are important in order to get physicians *to loosen up a bit and not be so uptight, get used to it and eventually embrace working in such a context.* It was important that physicians participating in the ERG were able to do exactly that, because they were entering a forum where an argument posed by an auxiliary nurse was as good as one posed by a senior physician.

A young physician explained that focusing on the ethics elements of daily practice was not something that she had been introduced to at university.

*Basically I don’t think we are very well prepared for what’s expected of us [ ]. That is actually the paradoxical thing about our education; you’re taught a number of specific professional skills, and all these other things, the things that connect them, the practical application, that’s something you’ll have to find out later. Perhaps it has to be like that, but the result is that we don’t have the tools to solve the problems, because solving problems is not like putting Lego bricks together, and that is mainly what we’ve been trained to do. I don’t think I would have found it strange if it [the ERG] wasn’t there. But now that I have tried it, I think it’s a good idea; it is in fact a part of what we do, that we have to consider the wider implications (Physician in individual interview).*

#### Challenges experienced by ethics facilitators

The abovementioned barriers related to clinicians also sometimes posed a challenge to the ethics facilitators. Some ethics facilitators worried that colleagues might challenge or question their skills and competences in ethics and ethics facilitation. One facilitator experienced such implicit criticism while managing an ERG.

*Another challenge we’ve come across, fortunately only once, was someone who rolled their eyes at most of what was going on [ ] Such a condescending attitude makes it difficult, it becomes a barrier to implementation, and a group of physicians who tend to agree that it’s all a waste of time, well, I think that makes it difficult to go in and start promoting ethics reflection, all geared up, telling people that oh this is so interesting and useful, when you can see that they’re thinking it’s boring and stupid (Ethics facilitator focus group).*

Some ethics facilitators experienced that participants with special authority or power sometime made their job difficult. In those situations, an atmosphere emerged which counteracted deliberation and dialog.

However, there were important examples of the opposite. One was a physician emphasizing that the other clinicians thought of him as “one of them”, and therefore they were neither afraid of him nor of participating in the dialog.

Participation by the ward managers varied across the three project sites. Most participants and ethics facilitators denied that management involvement posed a problem to the deliberation process. Yet some ethics facilitators experienced situations where the participation of superior staff could be a challenge.

*If participants who are also part of the management have a preferred direction they would like to pursue - well, they know the patients too, and then the discussion can easily end up going in that directions no matter what. Yes, and then there is a tendency that there’s one correct solution only. I’ve experienced that a couple of times (Ethics facilitator focus group).*

As can be seen above, there were both clinicians with special authority and ordinary staff among the participants in the ERG. The facilitators described how they felt a special responsibility towards ordinary clinicians finding themselves in an exposed situation, e.g. as the presenter of a case. One facilitator said that the presenter of a case might run the risk of concluding that he was responsible for doing something wrong, or alternatively unintentionally expose the actions of his colleagues.

#### New initiatives and changes based on the results and experiences of the study

After some working around the barriers, three ERGs were implemented. The outcome of the end-of-study workshop was an endorsement to continue the ERGs at all three project sites.

In the psychiatric department, there was a wish to establish even more groups. To meet the request to consolidate the ERGs at the hospital, some of the implementation strategies were changed. The one-day course for ethics facilitators continued, and a new network group was formed on the basis of the project group. The plan was for the group to meet for 3 hours every 6 months. The purpose of the network group was to anchor the ERGs in the hospital, and to provide a forum for ethics facilitators to exchange experiences, practice the SME model, and organize further education and training. Peer learning was planned to facilitate functioning across departments. Thus elements from the training and co-supervision implementation strategies were included in the network group. After project termination the network group had met two times, and the ERGs were organized as described in Table 6.

**Table 6:** Organization of ERGs at the termination of the research project

	Site I	Site IIB	Site IIB
Participants	Open interdisciplinary group	Open interdisciplinary group	Semi-open interdisciplinary group. The group had two permanent members from each of the three teams, and other clinicians participated ad hoc
Meeting place	Lunch room – experienced some disturbances	The group alternated between a meeting room without disturbances and the ward office with disturbances	A meeting room with no disturbances
Meeting time	Fixed meeting 45 min. every Tuesday morning	Ad hoc meetings of 45–60 min. once a month, organized to allow the afternoon shift to participate	Fixed meetings of 45 min. every second Wednesday morning
Participation of managers	Intermittent	Intermittent	Every meeting

## Discussion

Implementation of ERGs in hospital departments are complex interventions [35], influenced by many different factors. In this study, qualitative research methods have been used to identify barriers and promotors when implementing ERGs. The study reflects the experiences of various stakeholders and their perception of barriers and promotors found in relation to structural factors, but also in relation to clinicians and their pre-understandings, attitudes and interests in ERGs. To our knowledge, this is the first ERG implementation study to include both psychiatric and general hospital departments. In many ways, the findings in this research project strengthen and elaborate on previous findings from other contexts or other types of CESS, e.g. described by Haan et al. [24] and Meyer-Zehnder et al.. For Meyer-Zehnder et al. describe the following promotors: *acceptance and presence of the model, support given by the medical and nursing management, an existing or developing (explicit) ethics culture, perception of a need for a medical ethics decision-making model, and engaged staff members.* And they describe the following barriers: *Lack of presence and acceptance, insufficient time resources and staff, poor inter-professional collaboration, absence of ethical competence, and not recognizing ethical problems.*

### Promotors of the implementation of ethical reflection groups

#### Education and training of ethics facilitators and the usefulness of ethics reflection models

The education and training of ethics facilitators played a key role in implementation of the ERG. Introducing practical training for ethics facilitators in addition to the more theoretical courses helped the ethics facilitators to overcome some of their insecurity. The introduction of training in addition to teaching fits very well with literature on implementing MCD. Although the programs evaluated by Stolper et al. [28] are much more developed, they focus on new experimental learning paradigms,“learning by doing” and “reflection instead of ready-made knowledge” . They [28] conclude that their training program encouraged the participants to find their own style as ethics facilitators, without losing sight of the essence of MCD. In our project, over time the ethics facilitators became more accustomed to their role; identifying themselves as process managers, relaxing once they realized that the participants were to do the reflection; they were merely facilitators. When dialog got off track sometimes, the ethics facilitators found the SME model helpful for structuring and getting the focus back on the moral dimension. The use of a reflection method has been evaluated positively by others as well. Lillemoen et al. [10] find it an important success factor, helping the ethics facilitators to structure the discussion, and stick to the case.

#### Supportive ward managers

Like in other studies, having the support of the organization [24] is found to be an important promotor for the implementation of ERGs. The ward managers in this project supported the ERGs in different ways: One, for instance, participated in every meeting; another undertook the work duties of the participants while they attended the meeting. In this project, a cross-site project group served as a productive forum for managing different kinds of challenges, such as how to handle organizational obstacles or ethics facilitator insecurity. After project termination, this cross-site cooperation was continued in a network group in order to consolidate ERG in the organization.

#### Experience with ethics reflection among participants

Like in other studies [36], most clinicians welcomed ERG. Moreover, implementation was helped along by some prior knowledge about and experience with ethics reflection. Several years before the implementation of ERGs, a clinician at one of the project sites was involved in the CEC of Psychiatry, and she had arranged and facilitated meetings in the ward, using the SME model on ethics challenges experienced by clinicians in the ward. Some of the clinicians still remembered these meetings positively and said that the analysis had been useful to them. This clearly promoted recruitment of ethics facilitators and participants at this project site.

### Barriers to the implementation of ethical reflection groups

#### Lack of time

Lack of time constituted a barrier to the implementation of ERGs. Clinicians sometimes had to make difficult choices between attending to their patients and prioritizing participating in the ERG. If they chose to participate, their colleagues were left with more work. Lack of time also negatively influenced education and training of ethics facilitators. It was not possible for all ward managers to secure ethics facilitators time off to participate in the one-day course for ethics facilitators. Lack of time is a barrier found in other studies as well. Molewijk et al. [19] describe that newly trained ethics facilitators felt overburdened during the implementation process.

#### Administrative and financial management systems

Another barrier to implementation was financial management systems and administrative factors outside the control of the heads of departments; for instance, evaluation of the activities of individual clinicians based on number of patient consultations.

#### Preunderstandings about ethics

Also preunderstandings about ethics reflection among some clinicians acted as a barrier. Some were afraid the ERG would turn into an exclusive club; others thought ethics would only be about big and vague problems, or all talk and very little practical use.

#### Physicians and their attitudes towards ethical reflection seen in relation to the understanding of their job

Also, varying commitment and pre-understandings among physicians were found. One physician found the ERG to be useful as a way to introduce young physicians to an important part of clinical decision-making, while another was motivated by her fear of becoming a cynical physician. Besides, ethics reflection was also mentioned as something outside the scope of the job as a physician. Some physicians described themselves as “faultfinders” and “troubleshooters”, expected to have an answer to every question, leaving little room for doubt. Placed high in the hierarchy and responsible for the final decision, the role of a physician was described as opposed to engaging in a forum like an ERG, aspiring to be democratic. This description is underpinned by Hurst et al. investigating how physicians face ethical difficulties. Hurst et al. describe how physicians *sought to avoid conflict, obtain assistance, and protect the integrity of their conscience and reputation, as well as the integrity of the group of people who participated in the decisions* [37]*.* Also Agledahl et al. found physicians to have a way of dealing with ethical challenges that is not really compatible with inviting clinicians to take part in an interdisciplinary dialog. Investigating the moral practice of physicians from different practices, *it turned out that the doctors’ approach to clinical cases followed a rather strict pattern across specialties, which implied transforming patients’ diverse concerns into specific medical questions through a process of ‘essentialising’: Doctors broke the patient’s story down, concretised the patient’s complaints and categorised the symptoms into a medical sense* [38].

The theoretical foundation of systematic ethics reflection is hermeneutics [39] and discourse ethics [40]. Discourse ethics is characterized by Habermas’s ideal for ethical deliberations: *This principle is: ‘act only according to that norm of action that all rational beings and all those possibly affected by it can agree to in an unlimited (open), unconstrained rational discourse* [40]. *For the CEC, this translates into an ethical ideal of letting every stakeholder have his or her say, preferably by being present and taking part in the whole deliberations, or at least through a representative* [41]. This is the ideal of discourse ethics regarding moral issues, and this ideal is opposed to the belief that clinical decisions are based on medical facts only, or that good physicians has all the knowledge and answers needed.

Some of the barriers found among physicians in this study may be explained by physicians sometimes failing to acknowledge the needs for dialog between all involved parties to make good decisions.

### Strengths and limitations

One limitation of this study is that the ERGs were implemented in one country only. An important strength of the study is that the implementation strategies were used in both psychiatric and general hospital departments and in both in- and outpatient wards. Insights from all of these departments were homogeneous, which indicates a good external validity and applicability of the ERG implementation strategies in various hospital settings.

The individual interviews gave insight into the more personal values, attitudes and arguments for participation or non-participation in the ERGs. Using individual interviews, this study contributes to a clarification of the differences in physicians’ understandings of ethics in healthcare, and thus the study imparts new knowledge as to why physicians often decline to take part in ethics initiatives. This study includes a number of different data-gathering methods, but more extensive use of observational studies might have provided more information on how healthcare professionals actually acted during the implementation process. The research group took part in initiating, planning, performing and evaluating the study. This could be seen as a weakness as participants might be more reluctant to express any criticism. To overcome this, participants were actively encouraged to offer their own perspectives and critical comments, and during the individual interviews criticism was specifically asked for and examined.

## Conclusion

This study finds promotors of implementation to be: education and training of ethics facilitators, the usefulness of ethics reflection models, supportive ward managers, and experience with ethics reflection among participants. Also, this study finds barriers to implementation to be: lack of time, administrative and financial management systems, and preunderstandings about ethics. What this study adds is an elaboration on barriers related to physicians and their attitudes and interests in ethical reflection in relation to their understanding of their job. This study makes visible that by some the job of a physician might be described as opposed to engaging in a forum aspiring to be democratic, such as an ERG.

## Data Availability

In adherence with the regulations of the Danish Data Protection Agency and the period of time allowed for storage, data used and/or analyzed is available from the corresponding author on reasonable request.

## Abbreviations

CEC: Clinical ethics committee
CESS: Clinical ethics support service
ERG: ERG
MCD: Moral case deliberation
SME: “Senter for Medisinsk Etikk” in Norwegian; in English “Center for Medical Ethics”
